# CLINICAL FUNCTIONING INFORMATION TOOL – CORONAVIRUS DISEASE 2019 (CLINFIT COVID-19): PSYCHOMETRIC EVALUATION AND DEVELOPMENT OF AN INTERVAL-SCALED FUNCTIONING SCORE ACROSS THE CARE CONTINUUM

**DOI:** 10.2340/jrm.v57.43227

**Published:** 2025-08-17

**Authors:** Masahiko MUKAINO, Catarina AGUIARBRANCO, Alia ALGHWIRI, Sonia AMATO, Antonios KONTAXAKIS, Mihai Berteanu, Hüma BÖLÜK ŞENLIKCI, Pınar BORMAN, Salmane DIOUANE, Maryam FOURTASSI, Francesca GIMIGLIANO, Abderrazak HAJJIOUI, Xiaolei HU, Sinforian KAMBOU, Cho-I LIN, Mohamed I. MABROUK, Evanthia MITSIOKAPA, Remus Iulian NICA, Christina-Anastasia RAPIDI, Gabriella SERLENGA, Arianna SILVESTRI, Sinikka TARVONEN-SCHRÖDER, Clara URSESCU, Arja VIINANEN, Panagiotis VORNIOTAKIS, Melissa SELB

**Affiliations:** 1 Department of Rehabilitation Medicine I, School of Medicine, Fujita Health University, Toyoake; 2 Department of Rehabilitation Medicine, Hokkaido University Hospital, Sapporo, Japan; 3 Department of Physical and Rehabilitation Medicine, Centro Hospitalar de Entre o Douro e Vouga, Santa Maria da Feira; 4 Faculty of Dental Medicine, University of Porto, Portugal; 5 Department of Physiotherapy, School of Rehabilitation Sciences, The University of Jordan, Amman, Jordan; 6 Multidisciplinary Department of Medical-Surgical and Dental Specialities, University of Campania “Luigi Vanvitelli”, Naples, Italy; 7 Physical and Rehabilitation Medicine Department, 414 Military Hospital of Special Diseases, Athens, Greece; 8 University of Medicine and Pharmacy “Carol Davila” Bucharest, Romania; 9 University of Ankara Medipol, Faculty of Medicine, Department of Physical Medicine and Rehabilitation, Ankara, Turkey; 10 Faculty of Medicine and Pharmacy, University Sidi Mohammed Ben Abdellah, Fez, Morocco; 11 Life and Health Sciences Laboratory, Faculty of Medicine and Pharmacy, Abdelmalek Essaâdi University, Tangier, Morocco; 12 Department of Mental and Physical Health and Preventive Medicine, University of Campania “Luigi Vanvitelli“, Naples, Italy; 13 Department of Community Medicine and Rehabilitation, Umeå University, Sweden; 14 Institute of Applied Neurosciences and Functional Rehabilitation, Yaoundé, Cameroon; 15 Department of Physical Medicine and Rehabilitation, National Taiwan University Hospital Yunlin Branch, Yunlin, Taiwan; 16 Department of Physiotherapy, Faculty of Allied Medical Sciences, Applied Science Private University, Amman, Jordan; 17 Physical and Rehabilitation Medicine Department, Sotiria General Hospital of Chest Diseases of Athens, Greece; 18 Carol Davila University of Medicine and Pharmacy, Faculty of Midwifery and Nursing, Bucharest, Romania; 19 Physical and Rehabilitation Medicine Department, General Hospital “G.Gennimatas”, Athens, Greece; 20 Neurocenter, Turku University Hospital and Clinical Neurosciences, University of Turku, Turku, Finland; 21 Finnish Institute for Health and Welfare, Turku, Finland; 22 Department of Pulmonary Diseases, Turku University Hospital, Finland; 23 Pulmonary Diseases and Clinical Allergology, University of Turku, Finland; 24 Blocks Rehab, Rehabilitation Center, Athens, Greece; 26 ICF Research Branch, Nottwil, Switzerland; 27 Swiss Paraplegic Research, Nottwil, Switzerland

**Keywords:** COVID-19, functional status, International Classification of Functioning Disability and Health, outcomes, rehabilitation

## Abstract

**Objective:**

to report on the development and global testing of the COVID-19 version of the International Classification of Functioning, Disability and Health-based Clinical Functioning Information Tool called “ClinFIT COVID-19” to collect functioning data of rehabilitation patients across the care continuum to establish an interval-scaled functioning score.

**Design:**

Multicentre, cross-sectional observational study.

**Subjects/Patients:**

Rehabilitation patients in acute, post-acute, and long-term settings.

**Methods:**

Three context-specific versions (13–16 ICF categories) of ClinFIT-COVID-19 were administered to collect information on patient functioning. Rasch analysis examined psychometric properties and generated conversion tables from ordinal raw scores to a 0–100 interval metric.

**Results:**

Twenty-six study centres in 17 countries across the globe collected data from 1,747 patients. Problems in exercise tolerance functions were most frequently reported in the acute and post-acute settings (74.2%; 87.6%), while long-term care patients most frequently reported pain as problematic (71.1%). With a testlets approach and item splitting, all 3 ClinFIT COVID-19 versions satisfied Rasch model expectations (item-trait χ² *p* > 0.05; PSI 0.742–0.812), making it feasible to develop respective transformation tables.

**Conclusion:**

This study found the psychometric properties of ClinFIT COVID-19 acceptable. Future studies are needed to validate the use of the transformation tables to monitor functioning and evaluate intervention impact.

## LAY ABSTRACT

As COVID-19 symptoms, especially with long COVID, can significantly impact patients’ functioning and everyday life, conducting a comprehensive and holistic assessment is essential. This paper reports on the development and global testing of ClinFIT COVID-19, a clinical tool designed to collect information on functioning problems rehabilitation patients are experiencing, such as in energy level, physical exertion, self-care, moving around, etc. at different stages after a COVID infection. Other information, such as vaccination status, required hospitalizations, age, and gender were also collected. Statistical methods were used to analyse the tool’s measurement properties and to transform raw scores into the type of scores that can be used to compare patients and evaluate the effectiveness of an intervention. Twenty-six study centres in 17 countries across the globe collected data from 1,747 patients. Recently infected patients (4–12 weeks) experienced the most problems in fatigue, while pain was most frequently reported by long COVID patients.

Impacting millions of lives worldwide (1), the coronavirus disease 2019 (COVID-19) is associated with diverse symptoms due to multisystem effects, e.g., dyspnoea, fatigue, post-exertional malaise (PEM), palpitations, brain fog, and pain, which not only significantly impacted patients’ daily lives but also challenged health systems and society around the world (2–5). Post COVID-19 condition, as the World Health Organization (WHO) calls the condition with persistent late-onset symptoms, is estimated to cost society 1 trillion dollars annually and is a major issue to contend with in the management of COVID-19 (6, 7).

Despite the decreased incidence of COVID-19 (https://data.who.int/dashboards/covid19/cases) and symptom severity due to vaccination and virus transformation (8, 9), COVID-19 is still present, and the aforementioned COVID-19 symptoms can significantly impact patients’ functioning across the care continuum from acute to long-term. Functioning refers to a concept coined by WHO to represent the dynamic interaction between a health problem with the following dimensions of an individual’s health: physical and mental functions of the body (e.g., mental fatigue, exercise tolerance), body parts (e.g., lungs), ability to perform daily activities (e.g., self-care), participation at work, school or in the community, and environmental factors (e.g., medication) (10, 11). Given the impact of COVID-19 on functioning, ultimately diminishing the quality of life for patients, conducting a comprehensive assessment of functioning of COVID-19 patients that goes beyond the standard biomedically focused assessment, would be important (7). Such evaluations can provide a roadmap for targeted rehabilitative interventions, potentially optimizing recovery trajectories and enhancing patients’ everyday life.

Since 2019, the International Society of Physical and Rehabilitation Medicine (ISPRM) has been refining ClinFIT, a clinical functioning information tool based on WHO’s International Classification of Functioning, Disability, and Health (ICF) (10, 12). The standard ClinFIT is a generic tool based on the ICF Generic-30 Set, which encompasses 30 body function and activity/participation categories considered relevant across diverse health conditions (13). And in 2020, ISPRM started to develop a COVID-19 version of ClinFIT (14). ClinFIT COVID-19 is tailored to help healthcare professionals assess the multifaceted functioning issues of patients with COVID-19 in acute, post-acute, and long-term care settings.

The ClinFIT COVID-19 project also aimed to advance ClinFIT COVID-19 as an interval scale rather than as a conventional ordinal scale. Using an ordinal-scaled tool at 2 or more timepoints would enable the monitoring of changes in a patient’s functioning over time, indicating that a change (improvement, decline, or no change) has occurred. However, this change cannot be directly attributed to specific interventions, nor can the functioning status of a patient be compared with that of another patient. For this, ordinal-scaled patient data (as an overall functioning score) need to be transformed into interval-scaled scores. A Rasch-based methodology available for this transformation process (15, 16) requires collection of a certain amount and a broad spectrum of data representing the intended population.

Thus, the present study aimed to collect sufficient functioning data from patients all over the world and in various stages of care (acute, post-acute, and long-term) to enable the evaluation of ClinFIT COVID-19’s measurement properties in each care setting and to generate conversion tables that transform ordinal raw scores into interval-scaled values.

## METHODS

### Participants

Study centres (hospital/clinic, outpatient rehabilitation facility, or a rehabilitation provider in the community) in countries from all 6 WHO world regions were invited to participate in this cross-sectional study, utilizing ClinFIT COVID-19 to systematically collect functional information specifically focused on individuals diagnosed with COVID-19 (with signs and symptoms of COVID-19 < 12 weeks after onset) and post COVID-19 condition (defined in alignment with the WHO clinical case definition (17) as persistent signs and symptoms of COVID-19 that are not explained by an alternative diagnosis and that continue > 12 weeks after onset). The participants (also referred to as “patients”) were selected based on a convenience sample. Patients ≥ 18 years old with COVID-19 or post COVID-19 and able to understand the purpose of the study and to sign the informed consent form were included in the study. To avoid selection bias towards less severe patients, for individuals with severe symptoms or cognitive problems that prevented them from signing the consent form, family/caregiver with power of attorney was permitted to sign the consent form on behalf of the patient. Some countries refrained from this proxy option.

Each participating country was responsible for acquiring ethics approval based on the respective country requirements.

### Data collection

The participating countries employed different data collection methods, including paper-based, REDCap^®^ (https://project-redcap.org/), a secure web-based data management application provided by the main study centre Fujita Health University or another study centre-defined digital data collection platform. Data were submitted using one of 2 methods: (*i*) directly into an online REDCap database and (*ii*) via a standardized Excel sheet (Microsoft Corp, Redmond, WA, USA) sent to study centres that collected information on paper or via their own platforms. Irrespective of submission method, all the datasets were merged at the data centre (Fujita Health University), where they were cleaned and prepared for analysis.

Data were collected by rehabilitation physicians, physiotherapists, occupational therapists, speech therapists, or nurses. The health professionals involved in the study were offered a series of educational videos to ensure a comprehensive understanding of the study parameters and protocols, including a video explaining step-by-step how to use ClinFIT COVID-19 and how to collect the data.

Participating health professionals were asked to collect descriptive data, which comprised medical information (days after COVID-19 onset, vaccination status, rehabilitation service type, required hospitalization for COVID-19, ventilation support required due to COVID-19, current ventilation status, current need for assistance and mobility aids, pre-onset impairment), sociodemographic details (age, gender), and functioning data. The presence of PEM, a key functional symptom of post-COVID-19 condition, was recorded only for the long-term care patients. The functioning data were obtained using the ClinFIT COVID-19 for the acute, post-acute, and long-term care setting (14). The acute, post-acute, and long-term care versions were applied as follows: the acute care version covered the first 4 weeks or less, the post-acute care version was for the period more than 4 weeks up to 12 weeks, and the long-term care version was for periods extending beyond 12 weeks after the onset of COVID-19.

ClinFIT COVID-19 was developed following a multi-step process that began by identifying functioning aspects (ICF categories) that the tool should assess. The methodology for identifying the categories has already been described in a published article (14). The acute care version of ClinFIT COVID-19 consists of 13 items, the post-acute version 15 items, and the long-term version 16 items.

Each item is accompanied by a simple description, of which 13 have been published (16) and 4 (for the ICF categories b140, b440, b445, s430) were developed following the same established multi-step consensus process used to developed the published specifications with slight modifications to accommodate a virtual format (due to COVID measures at the time), see the Supplementary material.

ClinFIT COVID-19 offers 3 rating options:

0–4 scale where clinicians are asked to intuitively rate each item from 0: No problem to 4: Complete problem.

0–10 numeric rating scale (NRS) from 0: No problem to 10: Complete problem. Clinicians are familiar with using this option as the 0–10 NRS is commonly used in the evaluation of pain.

Mirrors the 0–4 rating option and additionally provides brief specifications for each response option. These specifications were originally developed in a project that implemented the ICF Generic-30 Set in Japan (19, 20).

Each rating sheet for the different ClinFIT COVID-19 versions and rating options can be found in the Supplementary material.

For the rating, clinicians are asked to consider the clinical meaning of the item according to the corresponding simple description and the results of routine clinical tools (e.g., physical examination, anamnesis, clinical tests, questionnaires).

### Data analysis

We employed Rasch analysis to evaluate the psychometric properties of ClinFIT COVID-19. It assesses item difficulty, i.e., how challenging each item is for respondents, and examines respondent ability levels, i.e., functioning level in this study. The Rasch model also enables the testing of fundamental measurement assumptions, such as the (*i*) model and item fit, (*ii*) reliability, (*iii*) unidimensionality of the scale, (*iv*) local independence of the items, and (*v*) the absence of subgroup effects on item difficulty, so-called differential item functioning (DIF). The latter ensures that the scale operates in the same way across different subgroups. If all the measurement assumptions are met, the Rasch analysis would support the internal construct validity of the instrument being tested, i.e., the extent the instrument itself measures the concept it claims to measure, as well as deliver a reliable interval-scaled score that can be used for the measurement of functioning.

In this study, the overall fit of the data to the Rasch model was investigated using χ^2^ statistics. A non-significant χ^2^ (> 0.05) value was interpreted as an overall good fit (21). For the items, a Bonferroni correction was applied to adjust for multiple testing, with an alpha cut-off of 0.05/n for n degrees of freedom. Reliability in this study is given with the Person Separation Index (PSI) with values 0.7–0.8 as acceptable, 0.8–0.9 as good reliability, and above 0.9 as very good reliability. Unidimensionality of the scale (i.e., that the scale measures a single underlying construct) was evaluated using the principal component analysis (PCA) (22). This assessment of unidimensionality involved conducting *t*-tests, which compared pairs of ability estimates derived from distinct Rasch calibrations of 2 item sets, categorized based on their positive or negative loadings on the first principal component of the PCA. The unidimensionality was indicated when the proportion of significant *t*-tests was below 5%. Regarding local independence (i.e., that the response to one item is not influenced by other items), a testlet approach was employed to improve the fit to the Rasch model when the local dependency of items interfered with item fit to the model (23). The testlets were developed by aggregating items that exhibit high residual correlations (i.e., response to 2 or more items is found to be related even after accounting for underlying traits) into “super-items” (aggregated items). In these super-items, an iterative process of scale adjustment, akin to that employed in single-item designs, was applied.

Lastly, regarding DIF, the lack of DIF is an important assumption in scale evaluation with the Rasch model (24, 25). The absence of DIF indicates that an individual can achieve comparable levels of ability regardless of group characteristics such as age and disease. In this study, DIF was investigated using an analysis of variance test for gender (male and female), age groups (< 30, 31–40, 41–50, 51–60, 61–70, and ≥ 71 years), disease groups (neurological, musculoskeletal, and others) and rating options (intuitive 0–4, intuitive 0–10, 0–4 with specifications). To resolve the DIF, item splitting, i.e., splitting an item that shows DIF into group-specific items, may improve the item fit (26).

In addition to investigating the fit to the Rasch model and the other measurement assumptions, this study also aimed to create a transformation table after a satisfactory fit to the Rasch model has been achieved. This transformation table transforms an ordinal-scaled score into an interval-scaled score. The analysis presenting the best fit with the Rasch model for each of acute, post-acute, and long-term care versions provided the foundation for this transformation table. This table was developed based on the estimates derived from the Rasch analysis.

All analyses were conducted using the statistical analysis software JMP 16 pro^®^ (https://www.jmp.com/en/software/predictive-analytics-software) and RUMM2030^®^ (https://www.rummlab.com.au/), the latter specifically designed for performing Rasch analyses.

## RESULTS

**Fig. 1:**
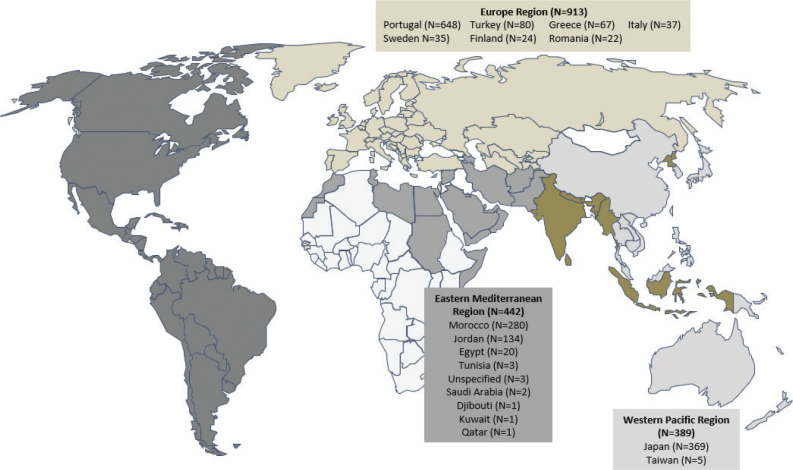
World map indicating the World Health Organization region in which the study centres (country) are located and amount of data collected.

Twenty-six centres across 17 countries in the WHO regions of Europe (Portugal, Greece, Sweden, Finland, Romania, Turkey, Italy), Eastern Mediterranean (Morocco, Jordan, Egypt, Tunisia, Saudi Arabia, Djibouti, Qatar and Kuwait) and Western Pacific (Japan, Taiwan) participated in this study (Table I and Fig. 1).

**Table I:** Distribution of cases by country and care setting

Country	Acute	Post-acute	Long-term	Scoring option
Djibouti	0	0	1	0–4 scale (with specifications)
Egypt	2	1	17	0–4 scale (with specifications)
Finland	9	1	14	0–4 scale (intuitive)
Greece	27	5	35	0–4 scale (intuitive)
Italy	7	8	22	0–4 scale (intuitive)
Japan	314	51	4	0–4 scale (with specifications)
Jordan	6	3	125	0–4 scale (with specifications)
Kuwait	0	0	1	0–4 scale (with specifications)
Morocco	9	15	256	0–4 scale (with specifications)
Portugal	13	162	473	0–4 scale (with specifications)
Qatar	0	0	1	0–4 scale (with specifications)
Romania	6	2	14	0–4 scale (with specifications)
Saudi Arabia	1	0	1	0–4 scale (intuitive)
Sweden	1	2	32	0–4 scale (intuitive)
Taiwan	2	7	11	0–4 scale (with specifications)
Tunisia	0	2	1	0–4 scale (intuitive)
Turkey	17	23	40	0–4 scale (intuitive)
Unspecified	1	0	2	0–4 scale (with specifications)
Total	415	282	1,050	

### Patient demographics

Data were collected from a total of 1,747 patients: 415 in acute care, 282 in post-acute care, and 1,050 in long-term care. Table II presents the results of sociodemographic and medical data collected. Overall, men and women were relatively equally represented (55.2% male), although the acute cohort contained more men (62.7%). Need for assistance with daily activities was most common in the post-acute group (51.1%), followed by the acute group (44.8%), and the long-term group (36.7%). Similarly, use of mobility aids was highest in the post-acute cohort (45.0%), compared with 19.0% in acute care, and 26.7% in long-term care. Noteworthy is the strong presence of PEM; among the 1,050 long-term care patients, 659 (62.8%) reported PEM, whereas 387 (36.9%) did not (4 values missing).

**Table II:** Results of sociodemographic and medical data collected

Item	Acute (*n* = 415)	Post-acute (*n* = 282)	Long-term (*n* = 1050)
Age, average (SD)	66.0 (19.8)	67.6 (21.3)	54.5 (19.4)
Gender, *n* (%)			
Male	260 (62.7%)	165 (58.5%)	539 (51.3%)
Female	155 (37.3%)	117 (41.5%)	511 (48.7%)
Days after onset, median (minimum/maximum)	11 (1/28)	33 (29/84)	372 (85/1080)
Hospitalization for COVID-19, *n* (%)	361 (87.0%)	229 (81.2%)	562 (53.5%)
Vaccination status, *n* (%)			
None	46 (11.08%)	17 (6.03%)	54 (5.14%)
Once	47 (11.33%)	12 (4.26%)	184 (17.52%)
Twice	126 (30.36%)	54 (19.15%)	457 (43.52%)
Three times or more	195 (46.99%)	198 (70.21%)	351 (33.43%)
Ventilation at any time due to COVID-19, *n* (%)			
Ventilation	33 (7.95%)	19 (6.74%)	277 (26.38%)
Oxygenation without ventilation	151 (36.39%)	183 (64.89%)	265 (25.24%)
None	231 (55.66%)	80 (28.37%)	508 (48.38%)
Ventilation at evaluation time, *n* (%)			
Ventilation	17 (4.10%)	0 (0%)	11 (1.05%)
Oxygenation without ventilation	91 (21.93%)	13 (4.61%)	11 (1.05%)
None	307 (73.98%)	269 (95.39%)	1,028 (97.90%)
Presence of PEM, *n* (%)			659 (62.76%)
Needs assistance from others, *n* (%)	186 (44.82%)	144 (51.06%)	385 (36.67%)
Needs mobility aids, *n* (%)	79 (19.04%)	127 (45.04%)	280 (26.67%)

SD: standard deviation; PEM: post-exertional malaise.

### Descriptive analysis

The analysis of functional problems of patients revealed significant variations in multiple ICF categories across the acute, post-acute, and long-term care context (see Table III). Specifically, in the acute patients, the ICF categories in which the most frequent problems (including mild to complete problems) were reported were b455 Exercise tolerance functions (74.2%), d230 Carrying out daily routine (64.6%), d450 Walking (61.0%), b440 Respiratory functions (59.8%), and b445 Respiratory muscle functions (54.5%). The post-acute care patients most frequently reported problems in b455 Exercise tolerance functions (87.6%), b730 Muscle power functions (85.1%), d455 Moving around (83.3%), b130 Energy and drive functions (81.9%), and b134 Sleep functions (82.3%). For the long-term care patients, the entity with most frequent problems was b280 Sensation of pain (71.1%); other frequent problems were in the entities b152 Emotional functions, b134 Sleep functions, b130 Energy and drive functions, d455 Moving around.

**Table III:** Percentage of patients and corresponding rating of functioning (per ICF category) in the acute, post-acute, and long-term context

	Acute						Post-acute						Long-term					
(%)	No problem	Mild problem	Moderate problem	Severe problem	Complete problem	Missing values	No problem	Mild problem	Moderate problem	Severe problem	Complete problem	Missing values	No problem	Mild problem	Moderate problem	Severe problem	Complete problem	Missing values
b130 Energy and drive functions	46.0	25.1	13.7	9.9	4.8	0.5	18.1	29.1	30.9	20.9	1.1	0.0	36.1	34.7	17.1	10.4	1.0	0.7
b134 Sleep functions	48.9	25.8	13.7	7.5	3.9	0.2	17.7	28.4	39.0	13.8	1.1	0.0	31.2	37.5	18.4	11.0	1.1	0.7
b140 Attention functions	59.5	15.2	8.7	7.7	4.1	4.8	18.1	8.2	62.8	3.5	0.0	7.4						
b152 Emotional functions	62.9	18.8	9.4	4.6	3.9	0.5	20.9	24.5	41.1	12.8	0.7	0.0	30.2	39.1	18.3	10.7	0.9	0.9
b280 Sensation of pain	67.7	18.3	9.9	2.4	1.4	0.2	20.6	28.7	46.5	3.5	0.7	0.0	28.4	33.9	30.6	5.4	1.2	0.5
b440 Respiratory functions	40.0	35.7	15.4	4.8	3.9	0.2	19.5	63.5	12.8	2.5	0.4	1.4	51.1	29.5	12.3	5.1	1.0	1.0
b445 Respiratory muscle functions	44.8	33.7	13.3	3.9	3.6	0.7	20.9	23.8	52.1	1.8	0.0	1.4	52.8	30.9	11.0	3.6	0.6	1.1
b455 Exercise tolerance functions	24.8	34.0	24.3	8.2	7.7	1.0	10.3	63.1	18.4	3.5	2.5	2.1	41.0	32.3	15.4	8.5	1.1	1.7
b710 Mobility of joint functions	64.8	20.2	8.4	3.9	2.2	0.5	20.9	30.1	38.7	9.2	0.7	0.4	51.9	21.2	15.2	9.5	1.2	0.9
b730 Muscle power functions	45.1	26.0	13.3	12.0	2.9	0.7	14.5	31.2	38.3	14.9	0.7	0.4	47.9	22.8	18.7	7.9	1.9	0.9
d230 Carrying out daily routine	35.4	15.9	14.7	10.6	23.4	0.0	18.8	25.9	39.4	10.6	5.3	0.0	53.2	23.1	15.8	6.3	1.0	0.6
d240 Handling stress and other psychological demands	55.9	25.5	7.7	4.3	6.5	0.0	18.1	35.5	28.4	16.3	1.8	0.0	51.4	20.3	20.3	6.2	1.0	0.9
d450 Walking	38.8	16.4	10.4	2.7	31.6	0.2	50.0	24.5	18.4	2.8	4.3	0.0	63.0	21.1	10.4	3.8	1.0	0.7
d455 Moving around							16.0	21.3	43.6	7.1	11.3	0.7	37.0	39.0	16.2	4.1	3.0	0.9
d850 Remunerative employment													29.9	35.9	6.7	3.4	2.4	21.8
d920 Recreation and leisure													52.8	28.2	11.2	4.4	1.9	1.5
s430 Structure of the respiratory system							16.0	10.3	63.1	4.6	0.4	5.7	51.8	28.8	12.6	2.1	1.1	3.6

### Use of ClinFIT COVID-19

Data from the majority of the 1,747 patients were collected in the long-term context (*n* = 1,050) using the corresponding version of ClinFIT COVID-19. The acute care version was used to collect the functioning data from 415 patients and the post-acute version from 282 patients. In terms of the rating options employed, none used the NRS option, while approximately 83% used the 0–4 scale with brief specifications for each response option, i.e., together with the acute care version for 346 patients, with the post-acute version for 234 patients, and with the long-term version for 882 patients.

### Rasch analysis

In this study, Rasch analysis was conducted on the acute, post-acute, and long-term care versions of ClinFIT COVID-19 (Table IV). Initially, the Rasch analysis revealed that the data from all 3 versions did not conform to the assumptions of the Rasch model. This was evident as the item-trait χ^2^ *p*-values were significant across all the analyses, indicating poor model fit. Additionally, issues of local dependencies, DIF, especially on age, were observed. To address these issues, a testlet approach was employed, where items were grouped into subgroups grouping items with high local dependency. When the testlet approach failed to resolve the DIF, item splitting was implemented as an alternative strategy. As the dataset included multiple age groups (< 30, 31–40, 41–50, 51–60, 61–70, and ≥ 71 years), item splitting by age was evaluated across various cutoff points, and the optimal solution was implemented.

**Table IV:** Summary of fit statistics for the acute, post-acute, and long-term versions of ClinFIT COVID-19 for the original scale, the testlet approach (aggregated), and the item split approach (by age)

Analysis	Location				Fit residual				Item–trait interaction			Reliability		Unidimensionality			CI% PST	LD	DIF
	Item		Persons		Item		Persons		χ^2^			PSI		Paired *t*-tests					
	Mean	SD	Mean	SD	Mean	SD	Mean	SD	Value	df	p	WITH extrms	NO extrms	N Significant tests	Sample	% PST			
Acute original	0.000	0.409	-1.103	1.107	-0.235	2.185	-0.298	1.269	354.6	117	0.000	0.811	0.812	40	387	10.3%	8.2-12.4%	Yes	Yes
2 testlets	0.000	0.053	-0.941	0.693	-0.544	2.223	-0.481	0.789	26.7	18	0.085	0.723	0.733	9	384	2.3%	0.2- 4.4%		Yes
2 testlets and item split with age (~60/61~)	0.000	0.186	-1.045	0.740	-0.564	1.405	-0.472	0.828	30.0	27	0.312	0.736	0.755						
2 testlets and item split with age (~60/61~), rating options (intuitive/with specifications)	0.000	0.190	-1.19	0.896	-0.351	1.163	-0.431	0.722	27.3	36	0.851	0.779	0.791						
Post-acute original	0.000	0.726	-1.254	1.338	-0.007	1.816	-0.796	1.931	573.9	135	0.000	0.897	0.865	47	269	17.5%	14.9 - 20.1%	Yes	Yes
3 testlets	0.000	0.306	-0.629	0.591	0.218	1.095	-0.637	1.099	27.5	27	0.434	0.717	0.660	17	254	6.7%	4.0-9.4%		Yes
3 testlets and item split with age (~70/71~)	0.000	0.300	-0.571	0.626	0.419	1.129	-0.589	1.078	32.1	36	0.655	0.725	0.641						
3 testlets and item split with age (~70/71~), rating options (intuitive/with specifications)	0.000	0.725	-0.899	0.636	0.544	0.865	-0.582	1.101	45.8	45	0.437	0.742	0.675						
Long-term original	0.000	0.260	-1.756	1.299	-0.551	3.511	-0.438	1.529	865.5	144	0.000	0.861	0.863	204	972	21.0%	19.6 - 22.4%	Yes	Yes
2 testlets	0.000	0.234	-1.128	1.028	-0.850	4.551	-0.472	0.608	41.2	18	0.001	0.804	0.805	28	752	3.7%	2.2 - 5.2%		Yes
2 testlets and item split with age (~30/31~)	0.000	0.194	-1.128	1.026	-0.752	3.401	-0.469	0.603	37.1	27	0.094	0.797	0.811						
2 testlets and item split with age (~30/31~), rating options (intuitive/with specifications)	0.000	0.292	-1.231	1.085	-0.298	3.026	-0.479	0.641	50.3	36	0.057	0.812	0.823						

df: degrees of freedom; extrms: extremes (extreme values); PSI: person separation index; % PST: percentage of significant *t*-tests, LD: local dependency, DIF: differential item functioning.

For the acute version, a 2-testlet approach was utilized, comprising motor/exercise tolerance and cognitive-related categories. As there was DIF in age and rating options, item splitting by age (60 or younger/older than 60) and rating options (intuitive 0–4/ 0–4 with specifications) was added. This approach demonstrated a good fit to the Rasch model, evidenced by non-significant item-trait χ^2^ *p*-value (0.851) and a PSI, an index for reliability for distinguishing between different levels of patient functioning, exceeding 0.7 (0.779; acceptable for group measurement).

Similar strategies were applied to the post-acute and long-term care versions. In the post-acute version, a 3-testlet approach (involving motor, exercise tolerance, and cognitive-related categories) with item split by age (70 or younger/older than 70) and rating options (intuitive 0–4/ 0–4 with specifications) also showed a good fit to the Rasch model, with non-significant item-trait χ^2^ *p*-value (0.437) and a person separation index exceeding 0.7 (0.742; acceptable for group measurement). Item splitting for the rating options was not required in the post-acute group under age 70 due to the absence of DIF. For the long-term care version, a 2-testlet approach with items (body function and activity/participation categories) split by age (under and over 30) and rating options (intuitive 0–4/ 0–4 with specifications) was adopted. This also resulted in an acceptable fit to the Rasch model with the non-significant item-trait χ^2^ *p*-value (0.057), and the PSI was above 0.8 (0.812).

Overall, all 3 versions of ClinFIT COVID-19 demonstrated acceptable psychometric properties after the application of testlet and item-splitting adjustments. Non-significant item–trait χ² statistics (*p* > 0.05) confirmed adequate model fit, and the PSI values indicated acceptable to good reliability for distinguishing levels of patient functioning. Unidimensionality, assessed via paired *t*-tests comparing subtests derived from the first principal component, showed fewer than 5% significant tests in the acute (2.3%) and long-term (3.7%) versions, and a marginally higher rate in the post-acute version (6.7%), though its 95% confidence interval (4.0–9.4%) includes the 5% threshold. Taken together, these findings support that each version measures a single underlying construct of functioning.

The transformation table of the ordinal-scaled raw scores into an interval-scale with 0–100 metric based on the results of the Rasch analysis is given in Table V.

**Table V:** Conversion table from raw score of ClinFIT COVID-19 to 0–100 interval scale

	Acute				Post-acute				Long-term				
	Age ≤60		Age >60			Age ≤70^*^	Age >70			Age ≤30		Age >30	
	Intuitive	With specifi-cations	Intuitive	With specifi-cations			Intuitive	With specifi-cations		Intuitive	With specifi-cations	Intuitive	With specifi-cations
0	0.0	0.0	0.0	0.0	0	0.0	0.0	0.0	0	0.0	0.0	0.0	0.0
1	6.4	5.9	7.2	7.6	1	5.1	6.0	7.0	1	9.5	8.5	9.7	6.8
2	10.5	9.8	12.0	12.9	2	7.9	8.9	11.4	2	15.6	13.8	16.6	11.6
3	13.2	12.5	15.2	16.7	3	9.4	10.3	14.1	3	19.6	17.0	21.7	14.9
4	15.2	14.4	17.7	19.8	4	10.5	11.0	15.9	4	22.7	19.4	25.8	17.6
5	16.9	16.0	19.8	22.4	5	11.4	11.5	17.1	5	25.2	21.3	29.4	20.0
6	18.2	17.3	21.5	24.6	6	12.2	11.8	18.1	6	27.4	22.9	32.5	22.1
7	19.4	18.3	23.1	26.5	7	12.9	12.1	18.8	7	29.4	24.4	35.3	24.2
8	20.4	19.1	24.6	28.2	8	13.6	12.4	19.6	8	31.2	25.7	37.7	26.0
9	21.3	19.8	25.9	29.6	9	14.2	12.6	20.2	9	32.8	27.0	39.8	27.8
10	22.1	20.4	27.0	30.8	10	14.9	12.8	20.8	10	34.4	28.3	41.7	29.5
11	22.8	21.0	28.1	31.9	11	15.5	13.0	21.5	11	35.8	29.4	43.4	31.0
12	23.4	21.5	29.0	32.8	12	16.2	13.2	22.1	12	37.2	30.5	45.0	32.4
13	23.9	21.9	29.9	33.7	13	16.8	13.4	22.7	13	38.6	31.6	46.5	33.8
14	24.5	22.4	30.7	34.5	14	17.4	13.6	23.4	14	39.9	32.6	47.9	35.0
15	24.9	22.8	31.4	35.2	15	18.0	13.8	24.1	15	41.1	33.5	49.2	36.1
16	25.3	23.2	32.1	35.9	16	18.6	14.0	24.8	16	42.3	34.4	50.4	37.2
17	25.7	23.6	32.8	36.5	17	19.2	14.2	25.4	17	43.4	35.3	51.6	38.1
18	26.1	24.0	33.4	37.1	18	19.8	14.5	26.1	18	44.5	36.1	52.8	39.0
19	26.5	24.5	34.0	37.7	19	20.3	14.7	26.7	19	45.6	36.8	53.9	39.8
20	26.9	24.9	34.6	38.3	20	20.8	15.0	27.3	20	46.6	37.4	54.9	40.5
21	27.3	25.3	35.2	38.8	21	21.4	15.2	27.9	21	47.6	38.0	55.9	41.1
22	27.6	25.8	35.7	39.3	22	21.9	15.5	28.5	22	48.6	38.6	56.9	41.6
23	28.0	26.2	36.3	39.9	23	22.5	15.7	29.1	23	49.6	39.1	57.8	42.1
24	28.5	26.7	36.9	40.4	24	23.1	16.0	29.8	24	50.5	39.5	58.7	42.5
25	28.9	27.2	37.4	40.9	25	23.7	16.2	30.4	25	51.4	40.0	59.5	42.9
26	29.4	27.6	38.0	41.5	26	24.3	16.5	31.2	26	52.2	40.3	60.3	43.2
27	29.9	28.1	38.7	42.0	27	25.0	16.7	32.0	27	53.1	40.7	61.1	43.6
28	30.5	28.6	39.3	42.6	28	25.7	17.0	32.9	28	53.9	41.1	61.8	43.9
29	31.2	29.1	40.0	43.2	29	26.6	17.3	33.9	29	54.7	41.4	62.5	44.2
30	31.9	29.7	40.7	43.8	30	27.4	17.6	35.1	30	55.5	41.8	63.2	44.5
31	32.7	30.3	41.5	44.4	31	28.3	18.1	36.7	31	56.3	42.1	63.9	44.8
32	33.6	30.9	42.3	45.1	32	29.2	18.6	38.1	32	57.0	42.4	64.5	45.0
33	34.7	31.6	43.3	45.9	33	30.2	19.7	38.8	33	57.8	42.8	65.1	45.3
34	35.9	32.3	44.3	46.7	34	31.2	20.4	39.3	34	58.5	43.1	65.6	45.6
35	37.2	33.2	45.5	47.5	35	32.2	20.7	39.6	35	59.2	43.5	66.1	45.8
36	38.7	34.1	46.7	48.5	36	32.9	20.9	39.9	36	60.0	43.8	66.7	46.1
37	40.3	35.1	48.1	49.5	37	33.5	21.0	40.2	37	60.7	44.2	67.2	46.4
38	42.1	36.3	49.6	50.7	38	33.9	21.2	40.4	38	61.4	44.7	67.6	46.7
39	44.1	37.5	51.3	51.9	39	34.2	21.3	40.6	39	62.1	45.1	68.1	46.9
40	46.3	39.0	53.2	53.3	40	34.5	21.3	40.8	40	62.8	45.6	68.6	47.2
41	48.7	40.6	55.2	54.8	41	34.8	21.5	41.0	41	63.5	46.0	69.0	47.5
42	51.3	42.5	57.3	56.5	42	35.1	21.5	41.2	42	64.2	46.5	69.5	47.8
43	54.2	44.6	59.7	58.4	43	35.3	21.6	41.4	43	64.9	47.1	69.9	48.1
44	57.2	47.3	62.2	60.5	44	35.5	21.7	41.6	44	65.7	47.6	70.4	48.4
45	60.5	50.6	65.0	63.0	45	35.7	21.8	41.9	45	66.4	48.1	70.9	48.7
46	64.1	54.7	68.0	65.8	46	36.0	21.9	42.1	46	67.2	48.7	71.3	49.1
47	68.0	59.8	71.1	69.2	47	36.2	22.0	42.4	47	67.9	49.3	71.8	49.5
48	72.4	65.9	74.6	73.2	48	36.4	22.2	42.8	48	68.8	49.9	72.3	49.9
49	77.4	73.0	78.6	77.9	49	36.8	22.6	43.2	49	69.6	50.5	72.9	50.3
50	83.2	81.0	83.4	83.4	50	37.0	25.8	43.7	50	70.4	51.2	73.4	50.8
51	90.7	89.9	90.3	90.6	51	37.4	27.8	44.3	51	71.3	51.8	74.0	51.4
52	100.0	100.0	100.0	100.0	52	37.9	29.9	45.2	52	72.2	52.5	74.6	52.0
					53	38.6	32.3	46.4	53	73.2	53.3	75.3	52.6
					54	44.0	34.9	48.4	54	74.2	54.1	76.0	53.4
					55	50.5	37.8	54.1	55	75.3	55.0	76.9	54.3
					56	57.7	41.1	61.1	56	76.5	56.0	77.7	55.4
					57	66.1	45.8	68.9	57	77.7	57.3	78.7	56.8
					58	75.9	55.1	77.9	58	79.1	58.8	79.9	58.6
					59	87.1	71.3	88.2	59	80.6	61.0	81.2	61.2
					60	100.0	100.0	100.0	60	82.4	64.2	82.9	65.0
									61	84.7	69.5	84.9	70.6
									62	87.6	77.1	87.8	78.2
									63	92.4	87.0	92.4	87.8
									64	100.0	100.0	100.0	100.0

* Note that item splitting for the rating options was necessary only in the group older than 70 due to the presence of DIF.

## DISCUSSION

In this study, we conducted an international pilot study using ClinFIT COVID-19, encompassing data from 1,747 patients across 17 countries to inform the development of an interval-scale score for ClinFIT COVID-19.

To our knowledge, ClinFIT COVID-19 is the first tool developed using WHO’s ICF framework to systematically capture and quantify COVID-19-related functioning across acute, post-acute, and long-term phases and piloted worldwide. This is particularly relevant in the context of COVID-19 and post-COVID-19 condition, where long-lasting impairments from the acute to long-term care context in physical, mental, and social functioning have been widely reported (27). The development of a structured, ICF-based tool to quantify functioning across these domains provides a much-needed foundation for assessing the trajectory of recovery and guiding individualized rehabilitation over time. The present study reached the intended aims, i.e., the collected data enabled us to examine the measurement properties of ClinFIT COVID-19 and to develop conversion tables that transformed ordinal raw scores into more nuanced interval-scaled values.

Rasch analysis of the data found that ClinFIT COVID-19’s psychometric properties are acceptable for clinical use. After adjusting the Rasch approach, data collected using the acute, post-acute, and long-term care versions of ClinFIT COVID-19 demonstrated a good fit to the Rasch model. The results of Rasch analysis enables the mapping of difficulty of individual items and identification of thresholds for different ratings, which, in turn, facilitates the development of an interval scale based on these results. An interval scale provides equal intervals between points, making it a more accurate measure of functioning ability compared with ordinal scales. The transformation table derived from the Rasch analysis allows for the conversion of ordinal raw scores into interval-scaled scores. The primary advantage of having an interval scale is that it allows for the measurement of change and differences with equal intervals between each score. When based on a well-fitting Rasch model and minimal bias, such interval-scaled scores support the quantification of patient functioning more effectively than ordinal scores. This consistency facilitates robust, linear measurement and can improve the interpretability of functioning assessments in both clinical and research settings (28). Such precision is particularly important in rehabilitation clinical practice, as it allows for reliable evaluation of intervention effects through pre–post or between-group analyses. From a clinical perspective, the use of interval scales enhances the interpretability of change (29). Many existing clinical assessments use ordinal scales, which indicate the order of performance levels but do not assume equal intervals between score points. As a result, mathematical operations such as calculating change scores or standard deviations are not appropriate. In contrast, interval scales assign equal value to each unit of change, enabling a clearer interpretation of the extent of improvement or deterioration. This, in turn, supports treatment goal-setting and development of therapeutic strategies. By providing a consistent and meaningful metric, interval scales facilitate more informed clinical decision-making, particularly when evaluating the effects of rehabilitation interventions across diverse settings. Moreover, interval-scaled scores can be employed for identifying classes of functioning trajectories (e.g., stable high functioning, early, moderate, and slow functioning improvement), that in turn, could support monitoring of outcomes of individual patients, benchmarking across patients, the development of patient pathways, and rehabilitation planning (30, 31). This approach may also enhance the accuracy of functioning prognosis, which is critical for designing efficient and targeted rehabilitation interventions.

The study also provided valuable insight into functioning in the acute, post-acute, and long-term context. In the acute care context, problems in exercise tolerance and respiratory-related functions were commonly observed. Interestingly, exercise tolerance problems were more frequently observed than respiratory problems, possibly due to the prevalence of fatigue independently of the respiratory issues in COVID-19 patients (32). This tendency was also present in the post-acute and long-term patients. PEM, i.e., extreme fatigue and flu-like symptoms disproportionate to a minimal physical or mental activity performed, which were frequently observed especially in the long-term patients (33), may have factored into this finding. Furthermore, the presence of myalgic encephalomyelitis/chronic fatigue syndrome has been found in over 50% of post-COVID-19 condition patients (32). Physical fatigue in the ICF (b4552 Fatiguability) is inherent in the ClinFIT COVID-19 item of exercise tolerance functions (10). On the other hand, mental fatigue (b1300 Energy level) is embedded in the ClinFIT COVID-19 item of energy and drive, and this was observed in all healthcare contexts. Although the energy and drive item does mention “psychological energy” in its description, this may not be understood as mental fatiguability. Furthermore, the exercise tolerance item does not refer to fatiguability at all in its description. Given this, the reporting of fatigue may have even been higher if these items were explicit about encompassing fatiguability. In any case, considering the high incidence of fatigue and PEM, the patient’s level of fatigue and presence of PEM should be considered when planning the intervention. The existing guidelines refer to energy conservation strategies or “pacing of activities”, which is to plan daily activities to manage the available energy, particularly for those in long-term care (7).

In terms of the high incidence of problems in d450 Walking in acute patients, this may be attributed to the necessity for bed-bound treatment, of which 87.0% was provided in hospitals. Furthermore, the quarantine restrictions aimed at preventing the spread of the disease may have also played a contributing role. Considering that reduced activity can precipitate functional decline, it is imperative to explore interventions, such as rehabilitation training programmes (e.g., muscle strength training and gait training) and a self-guided exercise programme, as well as environmental modifications, such as providing mobility aids and dedicated space for walking that would encourage body movement while accommodating the necessary treatment requirements.

### Limitations

There were several limitations in this study. First, there was a variation in patient clinical profiles across acute, post-acute, and long-term care settings that may have potentially led to selection bias. For example, 71.6% of post-acute patients experienced oxygenation including ventilation, compared with 44.3% of acute patients and 51.6% of long-term care patients. Furthermore, the study, primarily conducted in hospital settings, utilized a convenience sampling method; this may have influenced variation. For instance, post-acute patients who remain hospitalized generally had more severe symptoms compared with those in the acute phase. Therefore, the varying functioning profiles of patients make direct comparison across acute, post-acute, and long-term care settings more difficult. Moreover, patients with milder illness were potentially under-represented, as they were less likely to present to the hospital-based recruitment sites. Conversely, in countries that refrained from proxy consent, severely ill or cognitively impaired patients could not be enrolled, potentially leading to under-representation. This under-sampling may limit the generalizability of our findings to the full spectrum of COVID-19 and post-COVID-19 conditions. Demographic differences relative to other cohorts may reflect this skew. For example, while many post-COVID-19 cohorts are dominated by middle-aged women, our sample showed a more balanced gender distribution (34, 35). Nevertheless, the good sample size and the broad range of functioning data obtained within each setting still support the strength of the ClinFIT COVID-19.

Another potential limitation of this study is related to generalizability across country/regional, cultural, and economic resource contexts. Despite the participation of study centres in 17 countries across the globe, data were provided only from 3 WHO regions and primarily from high- and middle-resource countries. This can be partly explained by disease-reporting rates. More data collected in the WHO European region compared with the other regions seems to reflect the higher reporting rate of COVID-19 infections in Europe indicated on the WHO dashboard (https://data.who.int/dashboards/covid19/cases). In any case, pilot testing of ClinFIT COVID-19 in low-resource countries could help ensure that ClinFIT COVID-19 has utility across all cultural and economic resource contexts.

Additionally, to avoid potential bias associated with a skewed distribution of acute, post-acute, and long-term care patients across countries, regional differences are not discussed. Whether data were collected from acute, post-acute, or long-term patients was determined by the type of patients the participating hospitals and institutions predominately served. Including comparable hospitals and institutions in future research would enable reliable comparison of regional differences in COVID-19-related functional issues.

The lack of information on cognitive functions in the long-term care group may also be a limitation of this study. The decision on which items to include in each version of ClinFIT COVID-19 was based on a previous study conducted at the height of the COVID pandemic. Since then, post-COVID-19 condition has emerged, and recent cohort and survey studies report that 25–70% of individuals with post-COVID-19 condition experience persistent cognitive deficits – particularly in attention, memory, and executive functions – well beyond 12 weeks after infection (32, 33). Thus, the addition of cognitive functioning items may be considered in future versions of ClinFIT-COVID-19.

Similarly, it would be important to consider that patient pathways of COVID-19 and post-COVID-19 condition patients have evolved since the start of the COVID-19 pandemic. Thus, future studies may want to focus more on the functioning of patients in post-COVID condition and in the community as fatigue, cognitive impairments, pain, sleep problems, and psychological issues seem to persist 2 years post-infection (36).

### Conclusions

In this study, we conducted an international cross-sectional study using ClinFIT COVID-19, which revealed the spectrum of functioning problems encountered by COVID-19 and post-COVID-19 condition patients in acute, post-acute, and long-term contexts. Among the most prevalent issues identified were reduced exercise tolerance in the acute and post-acute phases and pain in long-term care, highlighting distinct patterns of functional impairment over time.

The Rasch analysis of the data supported the good psychometric properties of ClinFIT COVID-19 and enabled the development of an interval scale that transforms ordinal scores into a standardized interval-scaled metric. However, these findings provide only preliminary evidence for the potential utility of ClinFIT COVID-19. Further research is warranted to evaluate its practical value in clinical settings, such as for monitoring patient functioning or assessing the effects of interventions. Longitudinal studies are also needed to assess the instrument’s responsiveness to change and to establish minimal clinically important differences (MCIDs) for different patient populations.

In conclusion, this study represents an important first step toward the standardized assessment of patient functioning in COVID-19-related rehabilitation.

## Supplementary Material


