# Supplementary material for: CLINICAL FUNCTIONING INFORMATION TOOL – CORONAVIRUS DISEASE 2019 (CLINFIT COVID-19): PSYCHOMETRIC EVALUATION AND DEVELOPMENT OF AN INTERVAL-SCALED FUNCTIONING SCORE ACROSS THE CARE CONTINUUM
Source: J Rehabil Med. 2025 Aug 17;57:43227. doi: 10.2340/jrm.v57.43227 (PMC12375903; doi:10.2340/jrm.v57.43227)
Supplement: Supplementary file 1 [file JRM-57-43227-s1.pdf]

ClinFIT COVID-19 with 0–4 rating for acute settings

1	b130 Energy and drive functions<br>*Psychological energy and motivational drive to move towards goals, satisfy needs and control impulses*	**No problem**	**Mild**	**Moderate**	**Severe**	**Complete problem**	**Need more info**	**N/A**	**Pre-onset impairment?** **Yes** **No** **Unknown**
2	b134 Sleep functions<br>*Cycle, quality and amount of sleep*	**No problem**	**Mild**	**Moderate**	**Severe**	**Complete problem**	**Need more info**	**N/A**	**Pre-onset impairment?** **Yes** **No** **Unknown**
3	b140 Attention functions<br>*Focusing on needed information over a period of time*	**No problem**	**Mild**	**Moderate**	**Severe**	**Complete problem**	**Need more info**	**N/A**	**Pre-onset impairment?** **Yes** **No** **Unknown**
4	b152 Emotional functions<br>*Mental functions for the modulation of the expression of feelings and emotions*	**No problem**	**Mild**	**Moderate**	**Severe**	**Complete problem**	**Need more info**	**N/A**	**Pre-onset impairment?** **Yes** **No** **Unknown**
5	b280 Sensation of pain<br>*Unpleasant sensation indicating potential or actual damage of some body structure*	**No problem**	**Mild**	**Moderate**	**Severe**	**Complete problem**	**Need more info**	**N/A**	**Pre-onset impairment?** **Yes** **No** **Unknown**
6	b440 Respiratory functions<br>*Functions of breathing, including gas exchange*	**No problem**	**Mild**	**Moderate**	**Severe**	**Complete problem**	**Need more info**	**N/A**	**Pre-onset impairment?** **Yes** **No** **Unknown**
7	b445 Respiratory muscle functions<br>*Functions of the muscles involved in breathing*	**No problem**	**Mild**	**Moderate**	**Severe**	**Complete problem**	**Need more info**	**N/A**	**Pre-onset impairment?** **Yes** **No** **Unknown**
8	b455 Exercise tolerance functions<br>*Capacity of enduring physical exertion related to respiratory and cardiovascular functions*	**No problem**	**Mild**	**Moderate**	**Severe**	**Complete problem**	**Need more info**	**N/A**	**Pre-onset impairment?** **Yes** **No** **Unknown**
9	b710 Mobility of joint functions<br>*Range and ease of movement of a joint*	**No problem**	**Mild**	**Moderate**	**Severe**	**Complete problem**	**Need more info**	**N/A**	**Pre-onset impairment?** **Yes** **No** **Unknown**
10	b730 Muscle power functions<br>*Capacity to generate force through the contraction of a muscle or muscle groups*	**No problem**	**Mild**	**Moderate**	**Severe**	**Complete problem**	**Need more info**	**N/A**	**Pre-onset impairment?** **Yes** **No** **Unknown**
11	d230 Carrying out daily routine<br>*Plan, manage and complete routine daily life activities*	**No problem**	**Mild**	**Moderate**	**Severe**	**Complete problem**	**Need more info**	**N/A**	**Pre-onset impairment?** **Yes** **No** **Unknown**
12	d240 Handling stress and other psychological demands<br>*Manage and control the psychological demands to carry out tasks demanding responsibilities involving stress and/or distractions and/or critical issues*	**No problem**	**Mild**	**Moderate**	**Severe**	**Complete problem**	**Need more info**	**N/A**	**Pre-onset impairment?** **Yes** **No** **Unknown**
13	d450 Walking<br>*Moving in an upright position, step by step, always maintaining a support on the ground*	**No problem**	**Mild**	**Moderate**	**Severe**	**Complete problem**	**Need more info**	**N/A**	**Pre-onset impairment?** **Yes** **No** **Unknown**

ClinFIT COVID-19 with 0-10 Numeric Rating Scale for acute settings

1	b130 Energy and drive functions<br>*Psychological energy and motivational drive to move towards goals, satisfy needs and control impulses*	**No problem**	**0 1 2 3 4 5 6 7 8 9 10**	**Complete Problem**	**Need more info**	**N/A**	**Pre-onset impairment?**<br>**Yes No**<br>**Unknown**
2	b134 Sleep functions<br>*Cycle, quality and amount of sleep*	**No problem**	**0 1 2 3 4 5 6 7 8 9 10**	**Complete Problem**	**Need more info**	**N/A**	**Pre-onset impairment?**<br>**Yes No**<br>**Unknown**
3	b140 Attention functions<br>*Focusing on needed information over a period of time*	**No problem**	**0 1 2 3 4 5 6 7 8 9 10**	**Complete Problem**	**Need more info**	**N/A**	**Pre-onset impairment?**<br>**Yes No**<br>**Unknown**
4	b152 Emotional functions<br>*Mental functions for the modulation of the expression of feelings and emotions*	**No problem**	**0 1 2 3 4 5 6 7 8 9 10**	**Complete Problem**	**Need more info**	**N/A**	**Pre-onset impairment?**<br>**Yes No**<br>**Unknown**
5	b280 Sensation of pain<br>*Unpleasant sensation indicating potential or actual damage of some body structure*	**No problem**	**0 1 2 3 4 5 6 7 8 9 10**	**Complete Problem**	**Need more info**	**N/A**	**Pre-onset impairment?**<br>**Yes No**<br>**Unknown**
6	b440 Respiratory functions<br>*Functions of breathing, including gas exchange*	**No problem**	**0 1 2 3 4 5 6 7 8 9 10**	**Complete Problem**	**Need more info**	**N/A**	**Pre-onset impairment?**<br>**Yes No**<br>**Unknown**
7	b445 Respiratory muscle functions<br>*Functions of the muscles involved in breathing*	**No problem**	**0 1 2 3 4 5 6 7 8 9 10**	**Complete Problem**	**Need more info**	**N/A**	**Pre-onset impairment?**<br>**Yes No**<br>**Unknown**
8	b455 Exercise tolerance functions<br>*Capacity of enduring physical exertion related to respiratory and cardiovascular functions*	**No problem**	**0 1 2 3 4 5 6 7 8 9 10**	**Complete Problem**	**Need more info**	**N/A**	**Pre-onset impairment?**<br>**Yes No**<br>**Unknown**
9	b710 Mobility of joint functions<br>*Range and ease of movement of a joint*	**No problem**	**0 1 2 3 4 5 6 7 8 9 10**	**Complete Problem**	**Need more info**	**N/A**	**Pre-onset impairment?**<br>**Yes No**<br>**Unknown**
10	b730 Muscle power functions<br>*Capacity to generate force through the contraction of a muscle or muscle groups*	**No problem**	**0 1 2 3 4 5 6 7 8 9 10**	**Complete Problem**	**Need more info**	**N/A**	**Pre-onset impairment?**<br>**Yes No**<br>**Unknown**
11	d230 Carrying out daily routine<br>*Plan, manage and complete routine daily life activities*	**No problem**	**0 1 2 3 4 5 6 7 8 9 10**	**Complete Problem**	**Need more info**	**N/A**	**Pre-onset impairment?**<br>**Yes No**<br>**Unknown**
12	d240 Handling stress and other psychological demands<br>*Manage and control the psychological demands to carry out tasks demanding responsibilities involving stress and/or distractions and/or critical issues*	**No problem**	**0 1 2 3 4 5 6 7 8 9 10**	**Complete Problem**	**Need more info**	**N/A**	**Pre-onset impairment?**<br>**Yes No**<br>**Unknown**
13	d450 Walking<br>*Moving in an upright position, step by step, always maintaining a support on the ground*	**No problem**	**0 1 2 3 4 5 6 7 8 9 10**	**Complete Problem**	**Need more info**	**N/A**	**Pre-onset impairment?**<br>**Yes No**<br>**Unknown**

ClinFIT COVID-19 with 0-4 rating and specifications for each response item for acute settings

		0	1	2	3	4	N/A	Pre-onset impairment?	
1	b130 Energy and drive functions<br>*Psychological energy and motivational drive to move towards goals, satisfy needs and control impulses*	☐	☐	☐	☐	☐	☐	Yes ☐ No ☐<br>Unknown ☐	**0:** No problem<br>**1:** Mild problem: Patient has a problem but does not affect the patient's daily activities<br>**2:** Moderate problem: Patient has a problem that exceeds 1, but remains a relatively minor problem (<50%)<br>**3:** Severe problem: Patient has a major problem (≧ 50%)<br>**4:** Complete problem: Patient has a complete problem
2	b134 Sleep functions<br>*Cycle, quality and amount of sleep*	☐	☐	☐	☐	☐	☐	Yes ☐ No ☐<br>Unknown ☐	
3	b140 Attention functions<br>*Focusing on needed information over a period of time*	☐	☐	☐	☐	☐	☐	Yes ☐ No ☐<br>Unknown ☐	
4	b152 Emotional functions<br>*Mental functions for the modulation of the expression of feelings and emotions*	☐	☐	☐	☐	☐	☐	Yes ☐ No ☐<br>Unknown ☐	
5	b280 Sensation of pain<br>*Unpleasant sensation indicating potential or actual damage of some body structure*	☐	☐	☐	☐	☐	☐	Yes ☐ No ☐<br>Unknown ☐	
6	b440 Respiratory functions<br>*Functions of breathing, including gas exchange*	☐	☐	☐	☐	☐	☐	Yes ☐ No ☐<br>Unknown ☐	
7	b445 Respiratory muscle functions<br>*Functions of the muscles involved in breathing*	☐	☐	☐	☐	☐	☐	Yes ☐ No ☐<br>Unknown ☐	
8	b455 Exercise tolerance functions<br>*Capacity of enduring physical exertion related to respiratory and cardiovascular functions*	☐	☐	☐	☐	☐	☐	Yes ☐ No ☐<br>Unknown ☐	
9	b710 Mobility of joint functions<br>*Range and ease of movement of a joint*	☐	☐	☐	☐	☐	☐	Yes ☐ No ☐<br>Unknown ☐	
10	b730 Muscle power functions<br>*Capacity to generate force through the contraction of a muscle or muscle groups*	☐	☐	☐	☐	☐	☐	Yes ☐ No ☐<br>Unknown ☐	
11	d230 Carrying out daily routine<br>*Plan, manage and complete routine daily life activities*	☐	☐	☐	☐	☐	☐	Yes ☐ No ☐<br>Unknown ☐	**0:** No problem<br>**1:** Mild problem: Does the activity with assistive devices or does the activity with some difficulty<br>**2:** Moderate problem: Does the activity partially (less than 50%) with personal assistance<br>**3:** Severe problem: Does the activity largely (50% or more) with personal assistance<br>**4:** Complete problem: - Does the activity only with complete personal assistance. Otherwise, patient is unable to do the activity
12	d240 Handling stress and other psychological demands<br>*Manage and control the psychological demands to carry out tasks demanding responsibilities involving stress and/or distractions and/or critical issues*	☐	☐	☐	☐	☐	☐	Yes ☐ No ☐<br>Unknown ☐	
13	d450 Walking<br>*Moving in an upright position, step by step, always maintaining a support on the ground*	☐	☐	☐	☐	☐	☐	Yes ☐ No ☐<br>Unknown ☐	

ClinFIT COVID-19 with 0–4 rating for post-acute settings

1	b130 Energy and drive functions<br>*Psychological energy and motivational drive to move towards goals, satisfy needs and control impulses*	**No problem**	**Mild**	**Moderate**	**Severe**	**Complete problem**	**Need more info**	**N/A**	**Pre-onset impairment?**<br>**Yes** **No**<br>**Unknown**
2	b134 Sleep functions<br>*Cycle, quality and amount of sleep*	**No problem**	**Mild**	**Moderate**	**Severe**	**Complete problem**	**Need more info**	**N/A**	**Pre-onset impairment?**<br>**Yes** **No**<br>**Unknown**
3	b140 Attention functions<br>*Focusing on needed information over a period of time*	**No problem**	**Mild**	**Moderate**	**Severe**	**Complete problem**	**Need more info**	**N/A**	**Pre-onset impairment?**<br>**Yes** **No**<br>**Unknown**
4	b152 Emotional functions<br>*Mental functions for the modulation of the expression of feelings and emotions*	**No problem**	**Mild**	**Moderate**	**Severe**	**Complete problem**	**Need more info**	**N/A**	**Pre-onset impairment?**<br>**Yes** **No**<br>**Unknown**
5	b280 Sensation of pain<br>*Unpleasant sensation indicating potential or actual damage of some body structure*	**No problem**	**Mild**	**Moderate**	**Severe**	**Complete problem**	**Need more info**	**N/A**	**Pre-onset impairment?**<br>**Yes** **No**<br>**Unknown**
6	b440 Respiratory functions<br>*Functions of breathing, including gas exchange*	**No problem**	**Mild**	**Moderate**	**Severe**	**Complete problem**	**Need more info**	**N/A**	**Pre-onset impairment?**<br>**Yes** **No**<br>**Unknown**
7	b445 Respiratory muscle functions<br>*Functions of the muscles involved in breathing*	**No problem**	**Mild**	**Moderate**	**Severe**	**Complete problem**	**Need more info**	**N/A**	**Pre-onset impairment?**<br>**Yes** **No**<br>**Unknown**
8	b455 Exercise tolerance functions<br>*Capacity of enduring physical exertion related to respiratory and cardiovascular functions*	**No problem**	**Mild**	**Moderate**	**Severe**	**Complete problem**	**Need more info**	**N/A**	**Pre-onset impairment?**<br>**Yes** **No**<br>**Unknown**
9	b710 Mobility of joint functions<br>*Range and ease of movement of a joint*	**No problem**	**Mild**	**Moderate**	**Severe**	**Complete problem**	**Need more info**	**N/A**	**Pre-onset impairment?**<br>**Yes** **No**<br>**Unknown**
10	b730 Muscle power functions<br>*Capacity to generate force through the contraction of a muscle or muscle groups*	**No problem**	**Mild**	**Moderate**	**Severe**	**Complete problem**	**Need more info**	**N/A**	**Pre-onset impairment?**<br>**Yes** **No**<br>**Unknown**
11	d230 Carrying out daily routine<br>*Plan, manage and complete routine daily life activities*	**No problem**	**Mild**	**Moderate**	**Severe**	**Complete problem**	**Need more info**	**N/A**	**Pre-onset impairment?**<br>**Yes** **No**<br>**Unknown**
12	d240 Handling stress and other psychological demands<br>*Manage and control the psychological demands to carry out tasks demanding responsibilities involving stress and/or distractions and/or critical issues*	**No problem**	**Mild**	**Moderate**	**Severe**	**Complete problem**	**Need more info**	**N/A**	**Pre-onset impairment?**<br>**Yes** **No**<br>**Unknown**
13	d450 Walking<br>*Moving in an upright position, step by step, always maintaining a support on the ground*	**No problem**	**Mild**	**Moderate**	**Severe**	**Complete problem**	**Need more info**	**N/A**	**Pre-onset impairment?**<br>**Yes** **No**<br>**Unknown**
14	d455 Moving around<br>*Moving around differently from walking (for example running, going up and down the stairs, jumping, climbing, swimming, etc.)*	**No problem**	**Mild**	**Moderate**	**Severe**	**Complete problem**	**Need more info**	**N/A**	**Pre-onset impairment?**<br>**Yes** **No**<br>**Unknown**
15	s430 Structure of the respiratory system<br>*Trachea, lungs, ribcage, and breathing muscles*	**No problem**	**Mild**	**Moderate**	**Severe**	**Complete problem**	**Need more info**	**N/A**	**Pre-onset impairment?**<br>**Yes** **No**<br>**Unknown**

ClinFIT COVID-19 with 0-10 Numeric Rating Scale for post-acute settings

1	b130 Energy and drive functions<br>*Psychological energy and motivational drive to move towards goals, satisfy needs and control impulses*	**No problem** **0 1 2 3 4 5 6 7 8 9 10** **Complete Problem**	**Need more info**	**N/A**	**Pre-onset impairment?**<br>**Yes** **No**<br>**Unknown**
2	b134 Sleep functions<br>*Cycle, quality and amount of sleep*	**No problem** **0 1 2 3 4 5 6 7 8 9 10** **Complete Problem**	**Need more info**	**N/A**	**Pre-onset impairment?**<br>**Yes** **No**<br>**Unknown**
3	b140 Attention functions<br>*Focusing on needed information over a period of time*	**No problem** **0 1 2 3 4 5 6 7 8 9 10** **Complete Problem**	**Need more info**	**N/A**	**Pre-onset impairment?**<br>**Yes** **No**<br>**Unknown**
4	b152 Emotional functions<br>*Mental functions for the modulation of the expression of feelings and emotions*	**No problem** **0 1 2 3 4 5 6 7 8 9 10** **Complete Problem**	**Need more info**	**N/A**	**Pre-onset impairment?**<br>**Yes** **No**<br>**Unknown**
5	b280 Sensation of pain<br>*Unpleasant sensation indicating potential or actual damage of some body structure*	**No problem** **0 1 2 3 4 5 6 7 8 9 10** **Complete Problem**	**Need more info**	**N/A**	**Pre-onset impairment?**<br>**Yes** **No**<br>**Unknown**
6	b440 Respiratory functions<br>*Functions of breathing, including gas exchange*	**No problem** **0 1 2 3 4 5 6 7 8 9 10** **Complete Problem**	**Need more info**	**N/A**	**Pre-onset impairment?**<br>**Yes** **No**<br>**Unknown**
7	b445 Respiratory muscle functions<br>*Functions of the muscles involved in breathing*	**No problem** **0 1 2 3 4 5 6 7 8 9 10** **Complete Problem**	**Need more info**	**N/A**	**Pre-onset impairment?**<br>**Yes** **No**<br>**Unknown**
8	b455 Exercise tolerance functions<br>*Capacity of enduring physical exertion related to respiratory and cardiovascular functions*	**No problem** **0 1 2 3 4 5 6 7 8 9 10** **Complete Problem**	**Need more info**	**N/A**	**Pre-onset impairment?**<br>**Yes** **No**<br>**Unknown**
9	b710 Mobility of joint functions<br>*Range and ease of movement of a joint*	**No problem** **0 1 2 3 4 5 6 7 8 9 10** **Complete Problem**	**Need more info**	**N/A**	**Pre-onset impairment?**<br>**Yes** **No**<br>**Unknown**
10	b730 Muscle power functions<br>*Capacity to generate force through the contraction of a muscle or muscle groups*	**No problem** **0 1 2 3 4 5 6 7 8 9 10** **Complete Problem**	**Need more info**	**N/A**	**Pre-onset impairment?**<br>**Yes** **No**<br>**Unknown**
11	d230 Carrying out daily routine<br>*Plan, manage and complete routine daily life activities*	**No problem** **0 1 2 3 4 5 6 7 8 9 10** **Complete Problem**	**Need more info**	**N/A**	**Pre-onset impairment?**<br>**Yes** **No**<br>**Unknown**
12	d240 Handling stress and other psychological demands<br>*Manage and control the psychological demands to carry out tasks demanding responsibilities involving stress and/or distractions and/or critical issues*	**No problem** **0 1 2 3 4 5 6 7 8 9 10** **Complete Problem**	**Need more info**	**N/A**	**Pre-onset impairment?**<br>**Yes** **No**<br>**Unknown**
13	d450 Walking<br>*Moving in an upright position, step by step, always maintaining a support on the ground*	**No problem** **0 1 2 3 4 5 6 7 8 9 10** **Complete Problem**	**Need more info**	**N/A**	**Pre-onset impairment?**<br>**Yes** **No**<br>**Unknown**
14	d455 Moving around<br>*Moving around differently from walking (for example running, going up and down the stairs, jumping, climbing, swimming, etc.)*	**No problem** **0 1 2 3 4 5 6 7 8 9 10** **Complete Problem**	Need more info	**N/A**	**Pre-onset impairment?**<br>**Yes** **No**<br>**Unknown**
15	s430 Structure of the respiratory system<br>*Trachea, lungs, ribcage, and breathing muscles*	**No problem** **0 1 2 3 4 5 6 7 8 9 10** **Complete Problem**	Need more info	**N/A**	**Pre-onset impairment?**<br>**Yes** **No**<br>**Unknown**

ClinFIT COVID-19 with 0-4 rating and specifications for each response item for post-acute settings

	Item	0	1	2	3	4	N/A	Pre-onset impairment?	Rating specifications
1	b130 Energy and drive functions<br>*Psychological energy and motivational drive to move towards goals, satisfy needs and control impulses*	☐	☐	☐	☐	☐	☐	Yes ☐ No ☐<br>Unknown ☐	**0:** No problem<br>**1:** Mild problem: Patient has a problem but does not affect the patient's daily activities<br>**2:** Moderate problem: Patient has a problem that exceeds 1, but remains a relatively minor problem (<50%)<br>**3:** Severe problem: Patient has a major problem (≧ 50%)<br>**4:** Complete problem: Patient has a complete problem
2	b134 Sleep functions<br>*Cycle, quality and amount of sleep*	☐	☐	☐	☐	☐	☐	Yes ☐ No ☐<br>Unknown ☐	
3	b140 Attention functions<br>*Focusing on needed information over a period of time*	☐	☐	☐	☐	☐	☐	Yes ☐ No ☐<br>Unknown ☐	
4	b152 Emotional functions<br>*Mental functions for the modulation of the expression of feelings and emotions*	☐	☐	☐	☐	☐	☐	Yes ☐ No ☐<br>Unknown ☐	
5	b280 Sensation of pain<br>*Unpleasant sensation indicating potential or actual damage of some body structure*	☐	☐	☐	☐	☐	☐	Yes ☐ No ☐<br>Unknown ☐	
6	b440 Respiratory functions<br>*Functions of breathing, including gas exchange*	☐	☐	☐	☐	☐	☐	Yes ☐ No ☐<br>Unknown ☐	
7	b445 Respiratory muscle functions<br>*Functions of the muscles involved in breathing*	☐	☐	☐	☐	☐	☐	Yes ☐ No ☐<br>Unknown ☐	
8	b455 Exercise tolerance functions<br>*Capacity of enduring physical exertion related to respiratory and cardiovascular functions*	☐	☐	☐	☐	☐	☐	Yes ☐ No ☐<br>Unknown ☐	
9	b710 Mobility of joint functions<br>*Range and ease of movement of a joint*	☐	☐	☐	☐	☐	☐	Yes ☐ No ☐<br>Unknown ☐	
10	b730 Muscle power functions<br>*Capacity to generate force through the contraction of a muscle or muscle groups*	☐	☐	☐	☐	☐	☐	Yes ☐ No ☐<br>Unknown ☐	
11	d230 Carrying out daily routine<br>*Plan, manage and complete routine daily life activities*	☐	☐	☐	☐	☐	☐	Yes ☐ No ☐<br>Unknown ☐	**0:** No problem<br>**1:** Mild problem: Does the activity with assistive devices or does the activity with some difficulty<br>**2:** Moderate problem: Does the activity partially (less than 50%) with personal assistance<br>**3:** Severe problem: Does the activity largely (50% or more) with personal assistance<br>**4:** Complete problem: - Does the activity only with complete personal assistance. Otherwise, patient is unable to do the activity
12	d240 Handling stress and other psychological demands<br>*Manage and control the psychological demands to carry out tasks demanding responsibilities involving stress and/or distractions and/or critical issues*	☐	☐	☐	☐	☐	☐	Yes ☐ No ☐<br>Unknown ☐	
13	d450 Walking<br>*Moving in an upright position, step by step, always maintaining a support on the ground*	☐	☐	☐	☐	☐	☐	Yes ☐ No ☐<br>Unknown ☐	
14	d455 Moving around<br>*Moving around differently from walking (for example running, going up and down the stairs, jumping, climbing, swimming, etc.)*	☐	☐	☐	☐	☐	☐	Yes ☐ No ☐<br>Unknown ☐	
15	s430 Structure of the respiratory system<br>*Trachea, lungs, ribcage, and breathing muscles*	☐	☐	☐	☐	☐	☐	Pre-onset impairment?<br>Yes ☐ No ☐ Unknown ☐	

0: No problem: No abnormality in structure
1: Mild problem: Some abnormality, e.g. obsolete inflammatory changes without affecting function
2: Moderate problem: Partial abnormality (> 50%), e.g. lung fibrosis that affects function
3: Severe problem: Severe abnormality (<50%), e.g. marked atrophy of respiratory muscles that affects function
4: Complete problem: Abnormality in the whole structure, e.g. end-stage pulmonary fibrosis, that affects function

ClinFIT COVID-19 with 0–4 rating for long-term settings

1	b130 Energy and drive functions<br>*Psychological energy and motivational drive to move towards goals, satisfy needs and control impulses*	**No problem**	**Mild**	**Moderate**	**Severe**	**Complete problem**	**Need more info**	**N/A**	**Pre-onset impairment?** **Yes** **No** **Unknown**
2	b134 Sleep functions<br>*Cycle, quality and amount of sleep*	**No problem**	**Mild**	**Moderate**	**Severe**	**Complete problem**	**Need more info**	**N/A**	**Pre-onset impairment?** **Yes** **No** **Unknown**
3	b152 Emotional functions<br>*Mental functions for the modulation of the expression of feelings and emotions*	**No problem**	**Mild**	**Moderate**	**Severe**	**Complete problem**	**Need more info**	**N/A**	**Pre-onset impairment?** **Yes** **No** **Unknown**
4	b280 Sensation of pain<br>*Unpleasant sensation indicating potential or actual damage of some body structure*	**No problem**	**Mild**	**Moderate**	**Severe**	**Complete problem**	**Need more info**	**N/A**	**Pre-onset impairment?** **Yes** **No** **Unknown**
5	b440 Respiratory functions<br>*Functions of breathing, including gas exchange*	**No problem**	**Mild**	**Moderate**	**Severe**	**Complete problem**	**Need more info**	**N/A**	**Pre-onset impairment?** **Yes** **No** **Unknown**
6	b445 Respiratory muscle functions<br>*Functions of the muscles involved in breathing*	**No problem**	**Mild**	**Moderate**	**Severe**	**Complete problem**	**Need more info**	**N/A**	**Pre-onset impairment?** **Yes** **No** **Unknown**
7	b455 Exercise tolerance functions<br>*Capacity of enduring physical exertion related to respiratory and cardiovascular functions*	**No problem**	**Mild**	**Moderate**	**Severe**	**Complete problem**	**Need more info**	**N/A**	**Pre-onset impairment?** **Yes** **No** **Unknown**
8	b710 Mobility of joint functions<br>*Range and ease of movement of a joint*	**No problem**	**Mild**	**Moderate**	**Severe**	**Complete problem**	**Need more info**	**N/A**	**Pre-onset impairment?** **Yes** **No** **Unknown**
9	b730 Muscle power functions<br>*Capacity to generate force through the contraction of a muscle or muscle groups*	**No problem**	**Mild**	**Moderate**	**Severe**	**Complete problem**	**Need more info**	**N/A**	**Pre-onset impairment?** **Yes** **No** **Unknown**
10	d230 Carrying out daily routine<br>*Plan, manage and complete routine daily life activities*	**No problem**	**Mild**	**Moderate**	**Severe**	**Complete problem**	**Need more info**	**N/A**	**Pre-onset impairment?** **Yes** **No** **Unknown**
11	d240 Handling stress and other psychological demands<br>*Manage and control the psychological demands to carry out tasks demanding responsibilities involving stress and/or distractions and/or critical issues*	**No problem**	**Mild**	**Moderate**	**Severe**	**Complete problem**	**Need more info**	**N/A**	**Pre-onset impairment?** **Yes** **No** **Unknown**
12	d450 Walking<br>*Moving in an upright position, step by step, always maintaining a support on the ground*	**No problem**	**Mild**	**Moderate**	**Severe**	**Complete problem**	**Need more info**	**N/A**	**Pre-onset impairment?** **Yes** **No** **Unknown**
13	d455 Moving around<br>*Moving around differently from walking (for example running, going up and down the stairs, jumping, climbing, swimming, etc.)*	**No problem**	**Mild**	**Moderate**	**Severe**	**Complete problem**	**Need more info**	**N/A**	**Pre-onset impairment?** **Yes** **No** **Unknown**
14	d850 Remunerative employment<br>*Properly performing remunerative employment (full or part time or self-employed) in all its aspects*	**No problem**	**Mild**	**Moderate**	**Severe**	**Complete problem**	**Need more info**	**N/A**	**Pre-onset impairment?** **Yes** **No** **Unknown**
15	d920 Recreation and leisure<br>*Engaging in recreational or leisure activity (play, cultural & sports activities etc. during spare time)*	**No problem**	**Mild**	**Moderate**	**Severe**	**Complete problem**	**Need more info**	**N/A**	**Pre-onset impairment?** **Yes** **No** **Unknown**
16	s430 Structure of the respiratory system<br>*Trachea, lungs, ribcage, and breathing muscles*	**No problem**	**Mild**	**Moderate**	**Severe**	**Complete problem**	**Need more info**	**N/A**	**Pre-onset impairment?** **Yes** **No** **Unknown**

ClinFIT COVID-19 with 0-10 Numeric Rating Scale for long-term settings

1	b130 Energy and drive functions<br>*Psychological energy and motivational drive to move towards goals, satisfy needs and control impulses*	**No problem** 0 1 2 3 4 5 6 7 8 9 10 **Complete Problem**	**Need more info**	**N/A**	**Pre-onset impairment?** **Yes** **No** **Unknown**
2	b134 Sleep functions<br>*Cycle, quality and amount of sleep*	**No problem** 0 1 2 3 4 5 6 7 8 9 10 **Complete Problem**	**Need more info**	**N/A**	**Pre-onset impairment?** **Yes** **No** **Unknown**
3	b152 Emotional functions<br>*Mental functions for the modulation of the expression of feelings and emotions*	**No problem** 0 1 2 3 4 5 6 7 8 9 10 **Complete Problem**	**Need more info**	**N/A**	**Pre-onset impairment?** **Yes** **No** **Unknown**
4	b280 Sensation of pain<br>*Unpleasant sensation indicating potential or actual damage of some body structure*	**No problem** 0 1 2 3 4 5 6 7 8 9 10 **Complete Problem**	**Need more info**	**N/A**	**Pre-onset impairment?** **Yes** **No** **Unknown**
5	b440 Respiratory functions<br>*Functions of breathing, including gas exchange*	**No problem** 0 1 2 3 4 5 6 7 8 9 10 **Complete Problem**	**Need more info**	**N/A**	**Pre-onset impairment?** **Yes** **No** **Unknown**
6	b445 Respiratory muscle functions<br>*Functions of the muscles involved in breathing*	**No problem** 0 1 2 3 4 5 6 7 8 9 10 **Complete Problem**	**Need more info**	**N/A**	**Pre-onset impairment?** **Yes** **No** **Unknown**
7	b455 Exercise tolerance functions<br>*Capacity of enduring physical exertion related to respiratory and cardiovascular functions*	**No problem** 0 1 2 3 4 5 6 7 8 9 10 **Complete Problem**	**Need more info**	**N/A**	**Pre-onset impairment?** **Yes** **No** **Unknown**
8	b710 Mobility of joint functions<br>*Range and ease of movement of a joint*	**No problem** 0 1 2 3 4 5 6 7 8 9 10 **Complete Problem**	**Need more info**	**N/A**	**Pre-onset impairment?** **Yes** **No** **Unknown**
9	b730 Muscle power functions<br>*Capacity to generate force through the contraction of a muscle or muscle groups*	**No problem** 0 1 2 3 4 5 6 7 8 9 10 **Complete Problem**	**Need more info**	**N/A**	**Pre-onset impairment?** **Yes** **No** **Unknown**
10	d230 Carrying out daily routine<br>*Plan, manage and complete routine daily life activities*	**No problem** 0 1 2 3 4 5 6 7 8 9 10 **Complete Problem**	**Need more info**	**N/A**	**Pre-onset impairment?** **Yes** **No** **Unknown**
11	d240 Handling stress and other psychological demands<br>*Manage and control the psychological demands to carry out tasks demanding responsibilities involving stress and/or distractions and/or critical issues*	**No problem** 0 1 2 3 4 5 6 7 8 9 10 **Complete Problem**	**Need more info**	**N/A**	**Pre-onset impairment?** **Yes** **No** **Unknown**
12	d450 Walking<br>*Moving in an upright position, step by step, always maintaining a support on the ground*	**No problem** 0 1 2 3 4 5 6 7 8 9 10 **Complete Problem**	**Need more info**	**N/A**	**Pre-onset impairment?** **Yes** **No** **Unknown**
13	d455 Moving around<br>*Moving around differently from walking (for example running, going up and down the stairs, jumping, climbing, swimming, etc.)*	**No problem** 0 1 2 3 4 5 6 7 8 9 10 **Complete Problem**	Need more info	**N/A**	**Pre-onset impairment?** **Yes** **No** **Unknown**
14	d850 Remunerative employment<br>*Properly performing remunerative employment (full or part time or self-employed) in all its aspects*	**No problem** 0 1 2 3 4 5 6 7 8 9 10 **Complete Problem**	**Need more info**	**N/A**	**Pre-onset impairment?** **Yes** **No** **Unknown**
15	d920 Recreation and leisure<br>*Engaging in recreational or leisure activity (play, cultural & sports activities etc. during spare time)*	**No problem** 0 1 2 3 4 5 6 7 8 9 10 **Complete Problem**	Need more info	**N/A**	**Pre-onset impairment?** **Yes** **No** **Unknown**
16	s430 Structure of the respiratory system<br>*Trachea, lungs, ribcage, and breathing muscles*	**No problem** 0 1 2 3 4 5 6 7 8 9 10 **Complete Problem**	Need more info	**N/A**	**Pre-onset impairment?** **Yes** **No** **Unknown**

ClinFIT COVID-19 with 0-4 rating and specifications for each response item for long-term settings

	Item	0	1	2	3	4	N/A	Pre-onset impairment?	Rating
1	b130 Energy and drive functions<br>*Psychological energy and motivational drive to move towards goals, satisfy needs and control impulses*	☐	☐	☐	☐	☐	☐	Yes ☐ No ☐ Unknown ☐	**0:** No problem<br>**1:** Mild problem: Patient has a problem but does not affect the patient's daily activities<br>**2:** Moderate problem: Patient has a problem that exceeds 1, but remains a relatively minor problem (<50%)<br>**3:** Severe problem: Patient has a major problem (≧ 50%)<br>**4:** Complete problem: Patient has a complete problem
2	b134 Sleep functions<br>*Cycle, quality and amount of sleep*	☐	☐	☐	☐	☐	☐	Yes ☐ No ☐ Unknown ☐	
3	b152 Emotional functions<br>*Mental functions for the modulation of the expression of feelings and emotions*	☐	☐	☐	☐	☐	☐	Yes ☐ No ☐ Unknown ☐	
4	b280 Sensation of pain<br>*Unpleasant sensation indicating potential or actual damage of some body structure*	☐	☐	☐	☐	☐	☐	Yes ☐ No ☐ Unknown ☐	
5	b440 Respiratory functions<br>*Functions of breathing, including gas exchange*	☐	☐	☐	☐	☐	☐	Yes ☐ No ☐ Unknown ☐	
6	b445 Respiratory muscle functions<br>*Functions of the muscles involved in breathing*	☐	☐	☐	☐	☐	☐	Yes ☐ No ☐ Unknown ☐	
7	b455 Exercise tolerance functions<br>*Capacity of enduring physical exertion related to respiratory and cardiovascular functions*	☐	☐	☐	☐	☐	☐	Yes ☐ No ☐ Unknown ☐	
8	b710 Mobility of joint functions<br>*Range and ease of movement of a joint*	☐	☐	☐	☐	☐	☐	Yes ☐ No ☐ Unknown ☐	
9	b730 Muscle power functions<br>*Capacity to generate force through the contraction of a muscle or muscle groups*	☐	☐	☐	☐	☐	☐	Yes ☐ No ☐ Unknown ☐	
10	d230 Carrying out daily routine<br>*Plan, manage and complete routine daily life activities*	☐	☐	☐	☐	☐	☐	Yes ☐ No ☐ Unknown ☐	**0:** No problem<br>**1:** Mild problem: Does the activity with assistive devices or does the activity with some difficulty<br>**2:** Moderate problem: Does the activity partially (less than 50%) with personal assistance<br>**3:** Severe problem: Does the activity largely (50% or more) with personal assistance<br>**4:** Complete problem: - Does the activity only with complete personal assistance. Otherwise, patient is unable to do the activity
11	d240 Handling stress and other psychological demands<br>*Manage and control the psychological demands to carry out tasks demanding responsibilities involving stress and/or distractions and/or critical issues*	☐	☐	☐	☐	☐	☐	Yes ☐ No ☐ Unknown ☐	
12	d450 Walking<br>*Moving in an upright position, step by step, always maintaining a support on the ground*	☐	☐	☐	☐	☐	☐	Yes ☐ No ☐ Unknown ☐	
13	d455 Moving around<br>*Moving around differently from walking (for example running, going up and down the stairs, jumping, climbing, swimming, etc.)*	☐	☐	☐	☐	☐	☐	Yes ☐ No ☐ Unknown ☐	
14	d850 Remunerative employment<br>*Properly performing remunerative employment (full or part time or self-employed) in all its aspects*	☐	☐	☐	☐	☐	☐	Yes ☐ No ☐ Unknown ☐	
15	d920 Recreation and leisure<br>*Engaging in recreational or leisure activity (play, cultural & sports activities etc. during spare time)*	☐	☐	☐	☐	☐	☐	Yes ☐ No ☐ Unknown ☐	
16	s430 Structure of the respiratory system<br>*Trachea, lungs, ribcage, and breathing muscles*	☐	☐	☐	☐	☐	☐	Pre-onset impairment? Yes ☐ No ☐ Unknown ☐	

0: No problem: No abnormality in structure
1: Mild problem: Some abnormality, e.g. obsolete inflammatory changes without affecting function
2: Moderate problem: Partial abnormality (> 50%), e.g. lung fibrosis that affects function
3: Severe problem: Severe abnormality (<50%), e.g. marked atrophy of respiratory muscles that affects function
4: Complete problem: Abnormality in the whole structure, e.g. end-stage pulmonary fibrosi, that affects function
